# PTEN lipid phosphatase inactivation links the hippo and PI3K/Akt pathways to induce gastric tumorigenesis

**DOI:** 10.1186/s13046-018-0795-2

**Published:** 2018-08-22

**Authors:** Wenting Xu, Zhen Yang, Chuan Xie, Yin Zhu, Xu Shu, Zhe Zhang, Nianshuang Li, Na Chai, Song Zhang, Kaichun Wu, Yongzhan Nie, Nonghua Lu

**Affiliations:** 1 Department of Gastroenterology, The First Affiliated Hospital of Nanchang University, Nanchang, 330006 Jiangxi China; 2 State key Laboratory of Cancer Biology, National Clinical Research Center for Digestive Diseases and Xijing Hospital of Digestive Diseases, Fourth Military Medical University, Xi’an, China

**Keywords:** PTEN, Lipid phosphatase inactivation, Hippo, PI3K/Akt, Gastric cancer

## Abstract

**Background:**

Phosphatase and tensin homolog (PTEN) is an important tumor suppressor gene, and its encoded protein has activities of both a protein phosphatase and a lipid phosphatase. However, the substitution effect of protein phosphatase activity remains unclear. PI3K/Akt is the most common pathway negatively regulated by PTEN. The Hippo and PI3K/Akt pathways have a joint effect in regulating cell proliferation and apoptosis. Therefore, how PTEN lipid phosphatase inactivation contributes to the occurrence and development of gastric cancer and the potential role of the Hippo and PI3K/Akt pathways in PTEN lipid phosphatase inactivation mediated gastric tumorigenesis remain to be explored.

**Methods:**

Immunohistochemical staining was performed to detect the expression of p-PTEN and YAP in a gastric cancer tissue microarray. Stable cell lines expressing a wild-type or dominant-negative mutant PTEN were established. The proliferation and migration of stable cells were detected by MTT, BrdU, and colony-formation, transwell assay and high content analysis in vitro, and tumor growth differences were observed in xenograft nude mice. Changes in the expression of key molecules in the Hippo and Akt signaling pathways were detected by western blot. Nuclear-cytoplasm separation, immunofluorescence and coimmunoprecipitation analyses were conducted to explore the dysregulation of Hippo in the stable cell lines.

**Results:**

PTEN lipid phosphatase inactivation strongly promoted the proliferation and migration of gastric cancer cells in vitro and tumor growth in vivo. A immunohistochemical analysis of gastric cancer tissues revealed a significant correlation between phosphorylated PTEN and nuclear YAP expression, and both were determined to be independent prognostic factors for gastric cancer. Mechanistically, PTEN lipid phosphatase inactivation abolished the MOB1-LATS1/2 interaction, decreased YAP phosphorylation and finally promoted YAP nuclear translocation, which enhanced the synergistic effect of YAP-TEAD, thus inducing cell proliferation and migration. Moreover, PTEN lipid phosphatase inactivation promoted the PI3K/Akt pathway, and disruption of YAP-TEAD-driven transcription decreased the activation of Akt in a dose-dependent manner.

**Conclusions:**

Taken together, our findings indicate that PTEN lipid phosphatase inactivation links the Hippo and PI3K/Akt pathways to promote gastric tumorigenesis and cancer development.

**Electronic supplementary material:**

The online version of this article (10.1186/s13046-018-0795-2) contains supplementary material, which is available to authorized users.

## Background

Gastric cancer (GC) is the fourth most fatal malignant cancer worldwide and the second leading cause of cancer-related occurrence and death in China, which has had a severe impact on national health [1, 2]. Gastric carcinogenesis/development is a complicated multi-stage process, and during this process, the accumulation of genetic alterations plays an important role, such as the activation of an oncogene or the deletion of a tumor suppressor gene [3].

*Phosphatase and tensin homolog* (*PTEN*) is an important tumor suppressor gene that was discovered in 1997 [4]. Its encoded protein has activities of both a protein phosphatase and a lipid phosphatase and can regulate a complex network system dependent on phosphatase or non-phosphatase activity to affect biological functions [5–7]. The C124S mutation of PTEN can completely ablate its protein phosphatase and lipid phosphatase activity but does not affect its non-phosphatase function. The G129E mutation was discovered from a family with Cowden disease, and this mutation only abrogates its lipid phosphatase activity while maintaining its protein phosphatase activity [8, 9]. However, the substitution effect of protein phosphatase activity after the loss of lipid phosphatase activity remains unclear. Therefore, we investigated the effects of the loss of PTEN phosphatase function and the loss of its lipid phosphatase activity on GC.

The Hippo pathway is a highly conserved growth control signaling pathway identified in Drosophila in 1995 [10]. In recent years, the inactivation or abnormal regulation of Hippo has been found in lung adenocarcinoma, esophageal squamous cell carcinoma, liver cancer, ovarian serous cystadenoma, and bladder cancer [11–15]. The mammalian Hippo pathway is composed of three parts: multiple upstream signal input factors; the core kinase cascade reaction chain, including mammalian STE20-like protein kinase 1/2 (MST1/2), large tumor suppressor 1/2 (LATS1/2), human salvador homolog 1 (SAV1) and MOB kinase activator 1 (MOB1); and downstream transcription coactivators, including Yes-associated protein (YAP) and transcriptional coactivator with PDZ binding motif (TAZ) [16]. YAP and TAZ are the key effectors downstream of the pathway. YAP is localized at human chromosome 11q22 and can promote gene expression by enhancing transcription factor activity [17]. TAZ, located at chromosome 3q25, is homologous to YAP, and these proteins have similar functions [18]. Once Hippo has been activated by upstream signal molecules, MST1/2 binds to SAV1 and thus activates LATS1/2, which leads to YAP/TAZ phosphorylation and its stagnation in cytoplasm. If the pathway is blocked or inactivated, the nonphosphorylated YAP/TAZ will translocate into the nucleus. Because YAP/TAZ do not have DNA-binding sequences, they mainly combine with transcription factors in the nucleus, such as TEA domain family members (TEADs), to regulate the expression of downstream genes, inducing those related to cell transformation, proliferation, invasion and metastasis [19]. However, the exact regulatory mechanism of Hippo has not been fully explored and is still unclear.

At present, the most in-depth study of PTEN regards its negative regulation of the PI3K/Akt pathway in a phosphatase-dependent manner [20]. However, recent studies have found that PTEN can play a role that is independent of the PI3K/Akt pathway; therefore, the identification of other possible molecular mechanisms involved in PTEN function has important clinical significance [8, 21, 22]. Furthermore, the Hippo and PI3K/Akt signaling pathways have a joint effect in regulating cell proliferation and apoptosis [23]. How does PTEN phosphatase inactivation lead to the occurrence and development of GC? Are the Hippo and PI3K/Akt signaling pathways involved in PTEN phosphatase inactivation mediated gastric tumorigenesis? And what is the relationship between the Hippo and PI3K/Akt signaling pathways in this system? In view of these questions, this study investigated PTEN phosphatase inactivation and the PI3K/Akt and Hippo pathways and conducted in-depth studies at the human, animal and cell levels to provide new insights into the diagnosis and treatment of GC.

## Methods

### Tissue specimens

A GC tissue microarray containing 90 cases of GC and paired adjacent non-tumor tissues (ANTTs) was purchased from Shanghai Outdo Biotech (HStm-Ade180Sur-06). The study was approved by the Medical Research Ethics Committee and the Institutional Review Board of the First Affiliated Hospital of Nanchang University.

### Cell lines

BGC-823 and SGC-7901 cells were respectively cultured in Dulbecco’s Modified Eagle Medium and RPMI-1640 (Thermo Scientific HyClone, Beijing, China) supplemented with 10% fetal bovine serum (FBS), 100 U penicillin, and 100 μg/ml streptomycin (Gibco of Thermo Fisher Scientific Inc., Waltham, MA, USA) at 37 °C in an atmosphere of 5% CO_2_.

### Reagents and lentivirus

YAP-TEAD-driven transcription was disrupted with verteporfin (VP, SML0534; Sigma-Aldrich, St. Louis, MO, USA) [24]. The cells were incubated with different concentrations of VP for 72 h for cell quantification and motility detection. Wild-type PTEN, dominant-negative mutant type PTEN (C124S and G129E) and empty lentiviral supernatants were purchased from Invitrogen (of Thermo Fisher Scientific Inc.). For lentivirus infection, BGC-823 and SGC-7901 cells were grown to approximately 80% confluence and incubated with viral supernatants and hexadimethrine bromide (Sigma-Aldrich, St. Louis, MO, USA) for 6 h. Forty-eight hours later, the cells were split and cultured in selection media containing blasticidin (Sigma-Aldrich) for an additional 2 weeks. To isolate single cell lines, the cells were diluted and plated into wells at a ratio of 0.3 cells per well in 96-well plates in media without puromycin. Stable cell lines expressing wild-type or dominant-negative mutant PTEN were then established.

### Immunoblotting

Western blotting was performed according to standard method described previously [25] using the following antibodies: anti-PTEN (#9559; 1:1000), anti-phosphorylated (p)-PTEN (Ser380/Thr382/383) (#9554; 1:1000), anti-Akt (#4691; 1:1000), anti-p-Akt (Ser473) (#4060; 1:1000), anti-p-Akt (Thr308) (#13088; 1:1000), anti-Bad (#9239; 1:1000), anti-p-Bad (Ser136) (#4366; 1:1000), anti-p-FoxO1 (Ser256) (#9461; 1:1000), anti-FoxO1(#2880; 1:1000), anti-p27 Kip1 (#3686; 1:1000), anti-p-GSK-3β (#9323; 1:1000), anti-GSK-3β (#9315; 1:1000), anti-p-YAP (Ser 127) (#13008; 1:1000), anti-YAP/TAZ(#8418; 1:1000), anti-SAV1 (#13301; 1:1000), anti-LATS1 (#3477; 1:1000), anti-LATS2 (#5888; 1:1000), anti-MOB1 (#8699; 1:1000), anti-histone H3 (#4499; 1:1000), and anti-TEAD (#13295; 1:1000) from Cell Signaling Technology (Danvers, MA, USA) and anti-GAPDH (BA2913, 1:1000) from Boster Biological Technology (Preston, CA, USA).

### Immunohistochemistry

Immunohistochemistry was performed on 90 cases of GC and their paired ANTTs according to standard method described previously [25] using the following antibodies: anti-p-PTEN (Ser380/Thr382/383) (ab47332; 1:800) antibody (Abcam, Cambridge, UK), anti-YAP (#8418; 1:200) antibody and anti-p-Akt (Ser473) antibody (#4060; 1:100) (Cell Signaling Technology, Danvers, MA, USA). A total of 100 cells were counted in five random fields at 200×, and scores were evaluated based on the ratio and intensity of stained cells as described previously. Then, we categorized the expression into two groups based on score: score 0–3, low expression; score 4–12, high expression.

### MTT, BrdU, colony-formation, transwell assay and high content analysis

Cell survival was detected using the MTT assay. The stable cell lines expressing wild-type PTEN, dominant-negative mutant PTEN (C124S and G129E) or empty vector were seeded at a density of 2 × 10^3^ cells/well in a 96-well plate, and 20 μl of MTT solution (Sigma-Aldrich, St. Louis, MO, USA) was added 4 h before detection as described previously [20]. Cell proliferation was also detected using a BrdU assay kit (EMD Millipore, Billerica, MA, USA). For colony-formation assay, stable cell lines were trypsinized into a single-cell suspension and seeded in 6-cm dishes at 10^3^ per plate. After 2–3 weeks of incubation, the colonies were stained with crystal violet dye. For the migration assay*,* stable cell lines were seeded at a density of 3 × 10^4^ cells/well in transwell chambers (8.0-μm pore size; Corning Inc., Corning, NY, USA) with medium containing 20% FBS in the lower chamber as the chemoattractant and incubated for 48 h at 37 °C with 5% CO_2_. Cells that did not migrate through the pores were mechanically removed with a cotton swab. Cells attached to the bottom of the membrane insert were fixed in methanol at room temperature for 5 min and stained with hematoxylin. The number of invaded cells on the lower surface of the membrane was counted under a microscope at 400× magnification. For the high content analysis, stable cell lines were seeded at a density of 2 × 10^3^ cells/well in a 96-well plate, and after adherence, the cells were changed to medium without FBS and incubated overnight. The cells were then were placed into the high content analysis system to detect the cell numbers and relative motility distance.

### Mouse studies

Female BALB/c nude mice were provided by the Experimental Animal Center of the Fourth Military Medical University. All animals were housed and maintained in pathogen-free conditions. All animal studies complied with the Fourth Military Medical University animal use guidelines, and the protocol was approved by the Medical Research Ethics Committee and the Institutional Review Board of the First Affiliated Hospital of Nanchang University. Thirty-two nude mice were randomly divided into four groups according to the expression of the target genes: Control (with empty vector), WT (expressing WT PTEN), Mut 1 (expressing PTEN C124S), and Mut 2 (expressing PTEN G129E). The stable cell lines were trypsinized into a single-cell suspension and diluted to 1 × 10^7^/ml. A total of 200 μl of cells was injected subcutaneously into each flank of the nude mice. The tumor volumes were monitored using a living imaging system, and the growth curves of the tumors were plotted accordingly. After approximately 4 weeks, the nude mice were sacrificed, and the tumors were weighed.

### Coimmunoprecipitation

The appropriate cell numbers lysed for coimmunoprecipitation were determined by the same expression levels of LATS1 and LATS2 in the total lysate among groups. Coimmunoprecipitation was accomplished by incubating lysates with anti-MOB1 antibody (sc-161,867, Santa Cruz, Dallas, TX, USA) for 2 h and then with Protein A/G beads overnight. The immunoprecipitates were washed three times with lysis buffer. The immunoprecipitated proteins and input lysates were resolved by SDS-PAGE and then immunoblotted with the indicated antibodies.

### Immunofluorescence

The stable cell lines were seeded into an immunofluorescence culture chamber at the same density and cultured using standard protocols. The medium was removed, and the cells were washed with PBS and fixed in 4% formaldehyde solution for 15 min. The cells were then permeabilized in 0.5% Triton for 15 min, blocked with 10% serum for 30 min, and stained using indicated antibodies overnight at 4 °C. The cells were stained with the secondary antibody for 1 h and DAPI for 15 min and then imaged under a confocal microscope.

### Nuclear-cytoplasm separation

The nucleus-cytoplasm separation assay was performed using the Nucleus-cytoplasm Protein Extraction Kit (Beyotime Biotechnology, Shanghai, China). Extracted nuclear and cytoplasmic proteins were resolved by SDS-PAGE and then immunoblotted with the indicated antibodies.

### Statistical analyses

The data are summarized as the means±standard deviations (SDs) or percentages of the control. The chi-square test was performed to evaluate differences in categorical variables. One-way analysis of variance (ANOVA) was used to determine the differences in numerical variables. Kruskal-Wallis or Mann-Whitney tests were used to determine the differences in numerical variables between differently defined groups. Growth curves were plotted using the Kaplan-Meier method. An independent factor analysis for the prognosis of GC was performed using univariate and multivariate COX regression models; *p* < 0.05 was considered significant.

## Results

### P-PTEN is increased in GC tissues and correlates with clinicopathological characteristics of GC patients

**Fig. 1:**
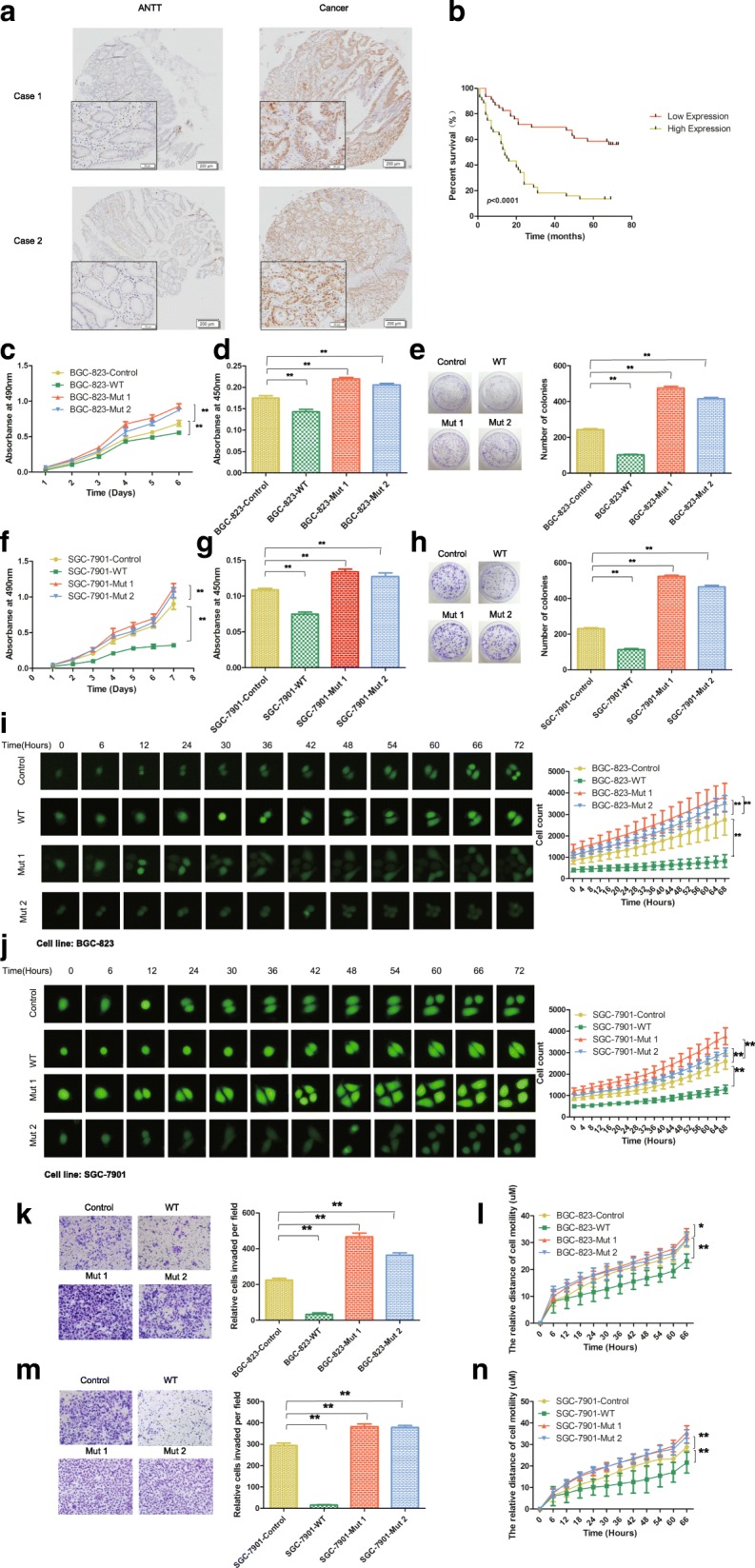
PTEN phosphatase inactivation is related to GC development. **a** IHC staining of p-PTEN in GC and ANTT tissues. **b** Kaplan–Meier analysis of p-PTEN expression and survival time in GC patients (*n* = 90; *p* < 0.05, log-rank test). **c**, **f** MTT assay, **d**, **g** BrdU assay, **e**, **h** colony-formation assay, and **i**, **j** high content analysis were performed to detect the proliferation of BGC823 and SGC7901 stable cells expressing wild-type PTEN, dominant-negative mutant type PTEN C124S (Mut 1) or G129E (Mut 2) or an empty vector. **k**, **m** Transwell and **l**, **n** high content analysis were conducted to detect the migration ability of the stable cells. The means±SEMs of a representative experiment (*n* = 3) performed in triplicate are shown. *, *p* < 0.05; **, *p* < 0.01

PTEN phosphorylation at Ser380/Thr382/Thr383 deprives PTEN of its phosphatase activity but maintains PTEN stability. Therefore, we evaluated the expression of p-PTEN (Ser380/Thr382/383) to observe the effect of PTEN phosphatase inactivation on gastric carcinogenesis in a cohort of 90 GC samples by immunohistochemistry. The results showed that p-PTEN was significantly upregulated in GC tissues compared with ANTTs (Fig. 1a; see also Additional file 1 for more detail). A correlation analysis showed that high expression of p-PTEN in GC was significantly associated with a more aggressive tumor phenotype, particularly T grade, N grade and clinical stage (Table 1). Furthermore, a Kaplan–Meier analysis indicated that high expression of p-PTEN was related to a shorter disease-free survival time for GC patients (Fig. 1b). Overall, these results indicated that an increase in p-PTEN with a loss of phosphatase activity is closely related to GC development and prognosis.

**Table 1:** Correlation of p-PTEN expression with clinicopathologic features in GC patients

Variables	p-PTEN expression			
	All cases (*n* = 90)	Low expression (*n* = 46)	High expression (*n* = 44)	*p* value
Sex				
Male	53	25	28	0.371
Female	37	21	16	
Age (years)				
≥ 63	46	22	24	0.822
< 63	44	24	20	
Tumor size (cm)				
≥ 5.5	42	19	23	0.297
< 5.5	48	27	21	
Location				
Upper 1/3	10	3	7	0.465
Middle	27	13	14	
Low 1/3	51	29	22	
Remnant	2	1	1	
T classification				
T1	3	3	0	0.019
T2	11	7	4	
T3	53	30	23	
T4	23	6	17	
N classification				
N0	22	21	1	0.000
N1/N2/N3	68	25	43	
Clinical stage				
I/II	36	32	4	0.000
III/IV	54	14	40	

### PTEN lipid phosphatase inactivation promotes GC proliferation and migration in vitro

To further investigate how PTEN phosphatase inactivation affects GC growth and invasion, we established stable cell lines that expressed a wild-type or dominant-negative mutant PTEN. PTEN was overexpressed in the stable cell line that carried the wild-type PTEN, which exhibited robust phosphatase activity. However, the C124S and G129E dominant-negative mutant forms of PTEN were catalytically dead forms of the phosphatase; the former mutant exhibits complete loss of phosphatase activity (Mut 1), whereas the latter only lacks lipid phosphatase activity (Mut 2). The empty vector did not carry any exogenous gene (Control). PTEN and p-PTEN were detected to confirm the establishment of these stable cells (see Additional file 2 for more detail). With regard to cell growth, MTT, BrdU and colony-formation assays revealed that PTEN lipid phosphatase inactivation significantly enhanced cell proliferation compared with the control, and the cell lines that carried the wild-type PTEN presented obviously decreased cell proliferation (Fig. 1c-h). In addition, the high content analysis showed increases in the cell division rate and total cell numbers in the Mut 1 and Mut 2 groups, whereas a decrease in these parameters was found in the WT group (Fig. 1i, j). With regard to cell migration, a transwell analysis showed that the dominant-negative mutant forms of PTEN significantly upregulated cell migration, whereas the WT group exhibited reduced migration compared with the control (Fig. 1k, m). Furthermore, the high content analysis revealed that PTEN lipid phosphatase inactivation increased the mean cell migration distance (Fig. 1l, n). Overall, these results suggested an important role for PTEN lipid phosphatase inactivation in GC development in vitro.

### PTEN lipid phosphatase inactivation promotes tumor growth in nude mice

**Fig. 2:**
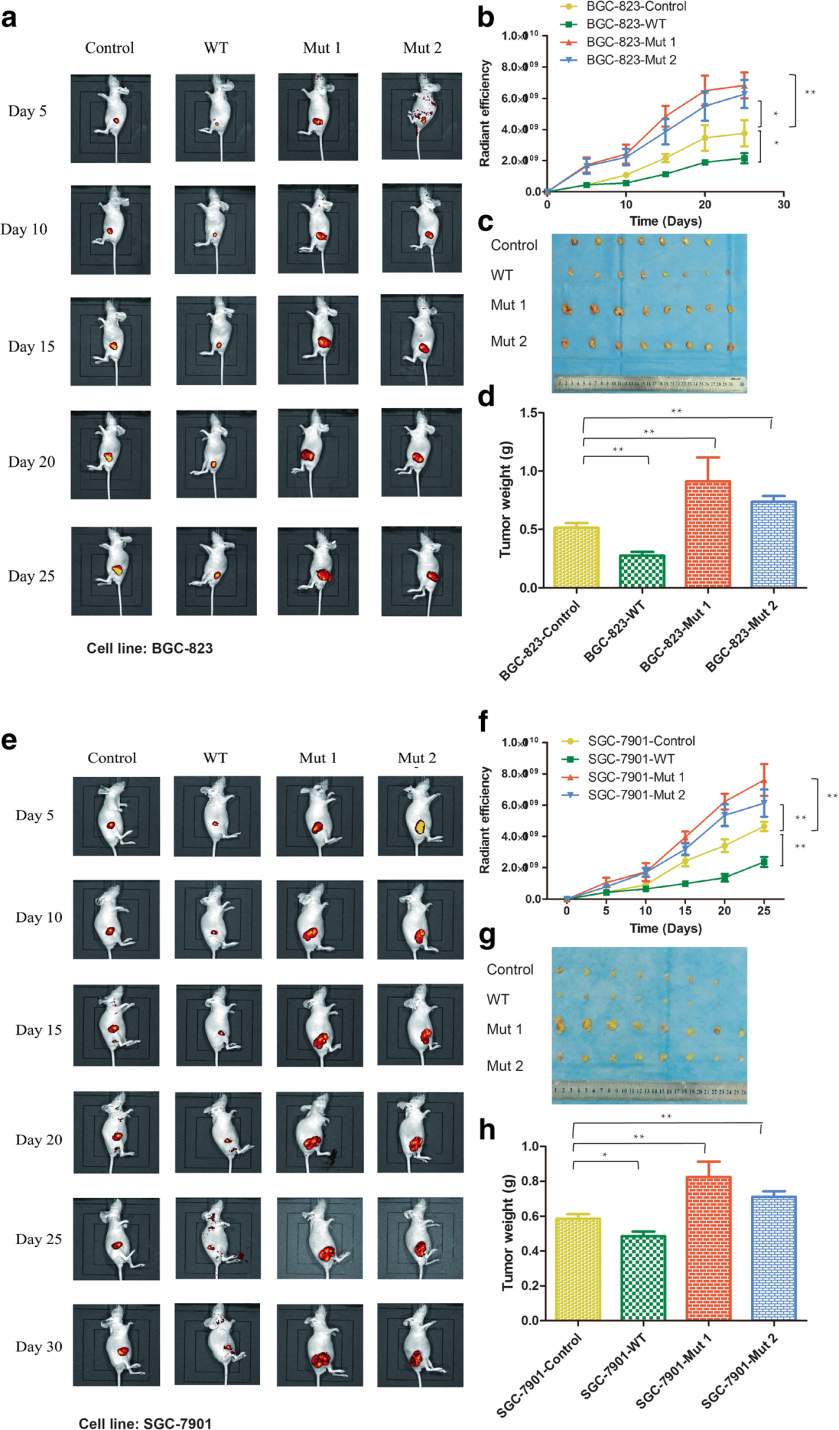
PTEN lipid phosphatase inactivation promotes tumor growth in nude mice BGC823 and SGC7901 stable cells infected with an empty vector, wild-type PTEN, or dominant-negative mutant type PTEN C124S (Mut 1) or G129E (Mut 2) lentiviral were injected subcutaneously into nude mice. **a**, **e** Representative live imaging of tumors formed in nude mice at days 5, 10, 15, 20, 25 or 30 (*n* = 8) and **b**, **f** quantification of tumor growth curves. **c**, **g** All tumors isolated from the mice. **d**, **h** quantification of tumor weight *, *p* < 0.05; **, *p* < 0.01.

To identify the role of PTEN lipid phosphatase inactivation in gastric tumorigenesis in vivo, a xenograft model was adopted using nude mice. Stable cell lines expressing wild-type PTEN, dominant-negative mutant PTEN (C124S and G129E) or an empty vector were injected subcutaneously into each flank of nude mice. The results showed that PTEN lipid phosphatase inactivation significantly increased the tumor growth rate, tumor volume and tumor weight in vivo (Fig. 2a–h). Overall, these results indicated that PTEN lipid phosphatase inactivation affected GC development in vivo.

### The YAP level is positively correlated with GC progression and the p-PTEN level

**Fig. 3:**
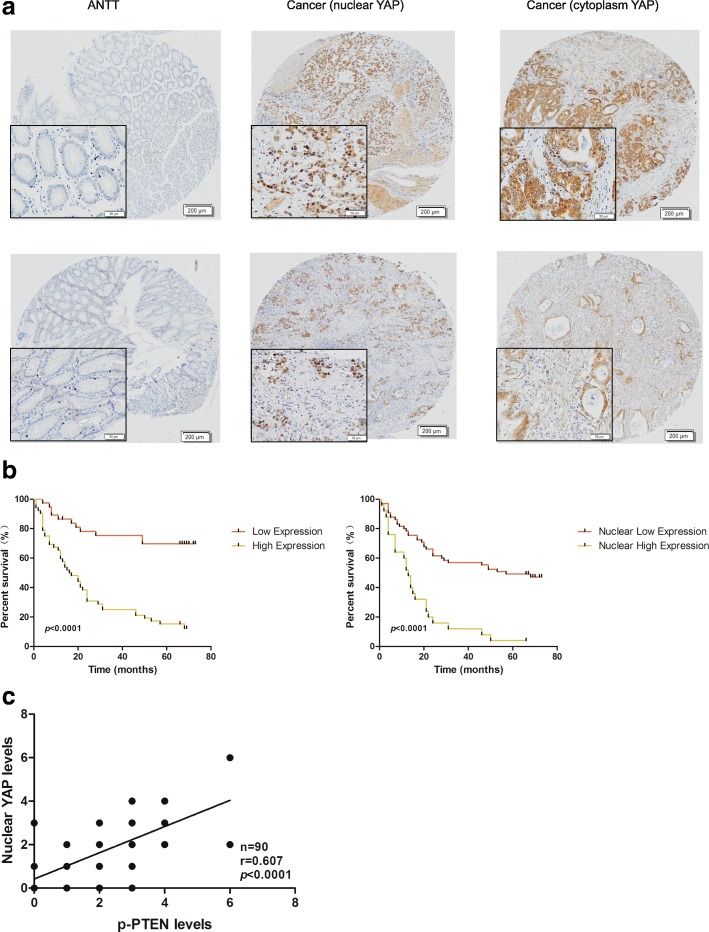
The YAP level is positively correlated with the progression of GC and p-PTEN level. **a** Representative images of IHC staining for YAP in a GC tissue microarray. **b** Kaplan–Meier analysis of total YAP and nuclear YAP expression with survival time in GC patients. **c** Association between p-PTEN expression and nuclear YAP levels in GC specimens

YAP is the key effector downstream of the Hippo pathway. The YAP level in the cohort of 90 GC samples indicated previously was detected by IHC. Compared with the ANTTs, the YAP level was increased in GC tissues (Fig. 3a; see Additional file 3 for more detail). The correlation analysis showed that high expression of YAP and nuclear YAP were significantly associated with a more aggressive tumor phenotype in GC, such as tumor size, T grade, N grade and clinical stage (Table 2). Furthermore, the Kaplan–Meier analysis indicated that high expression of YAP and nuclear YAP were related to a shorter disease-free survival time for GC patients (Fig. 3b). The correlation between p-PTEN and nuclear YAP expression in 90 cases of GC was analyzed. It showed that p-PTEN expression was positively correlated with expression of nuclear YAP (Fig. 3c). A Cox regression analysis also showed that high expression of phosphorylated PTEN, YAP, and nuclear YAP were independent prognostic factors for poor survival in GC patients (Table 3).

**Table 2:** Correlation of YAP protein expression with clinicopathologic features in GC patients

Variables	YAP expression									
	All cases (*n* = 90)	Low expression (*n* = 37)	High expression (*n* = 53)	*p* value	High nuclear expression (*n* = 25)	Low nuclear expression (*n* = 65)	*p* value	High cytoplasmic expression (*n* = 21)	Low cytoplasmic expression (*n* = 69)	*p* value
Sex										
Male	53	22	31	0.927	15	38	0.894	13	40	0.748
Female	37	15	22		10	27		8	29	
Age (years)										
≥ 63	46	19	27	0.970	12	34	0.714	11	35	0.894
< 63	44	18	26		13	31		10	34	
Tumor size (cm)										
≥ 5.5	42	12	30	0.024	16	26	0.041	11	31	0.549
< 5.5	48	25	23		9	39		10	38	
Location										
Upper 1/3	10	3	7	0.424	3	7	0.492	3	7	0.518
Middle	27	11	16		10	17		4	23	
Low 1/3	51	22	29		12	39		13	38	
Remnant	2	1	1		0	2		1	1	
T classification										
T1	3	3	0	0.040	0	3	0.003	0	3	0.708
T2	11	5	6		3	8		2	9	
T3	53	24	29		9	44		14	39	
T4	23	5	18		13	10		5	18	
N classification										
N0	22	17	5	0.000	1	21	0.005	4	18	0.511
N1/N2/N3	68	20	48		24	44		17	51	
Clinical stage										
I/II	36	27	9	0.000	2	34	0.000	6	30	0.222
III/IV	54	10	44		23	31		15	39

**Table 3:** Summary of univariate and multivariate Cox regression analysis of overall survival duration

Parameter	Case number	*p*	HR	95% CI
Univariate analysis				
Sex (Male vs. Female)	53/37	0.149	0.67	0.39–1.15
Age (≥63 vs. < 63)	46/44	0.0673	0.61	0.36–1.04
Tumor size (≥5.5 vs. < 5.5)	42/48	0.1179	1.527	0.90–2.60
Location (Upper vs. Middle vs. Low vs. Remnant)	10/27/51/2	0.3653	–	–
T classification (T1/T2 vs. T3/T4)	14/76	0.0031	0.39	0.21–0.73
N classification (No vs. N1/N2/N3)	22/68	< 0.0001	0.26	0.15–0.45
YAP (Low vs. High)	37/53	< 0.0001	0.23	0.14–0.40
YAP (Nuclear high vs. Nuclear low)	25/65	< 0.0001	0.17	0.09–0.35
YAP (Cytoplasm high vs. Cytoplasm low)	69/21	0.4020	0.77	0.41–1.43
p-PTEN (Score: 0 vs. 1 vs. 2 vs. 3 vs. 4 vs. 5 vs. 6)	10/5/31/36/6/0/2	< 0.0001	–	–
Multivariate analysis				
T classification (T1/T2 vs. T3/T4)	14/76	0.043	2.92	1.03–8.28
N classification (No vs. N1/N2/N3)	22/68	0.004	4.66	1.61–13.46
YAP (Low vs. High)	37/53	0.001	3.70	1.70–8.04
YAP (Nuclear high vs. Nuclear low)	25/65	0.028	1.98	1.07–3.63
p-PTEN (Score: 0 vs. 1 vs. 2 vs. 3 vs. 4 vs. 5 vs. 6)	10/5/31/36/6/0/2	0.045	0.80	0.65–1.00

### PTEN lipid phosphatase inactivation negatively regulates the hippo pathway

**Fig. 4:**
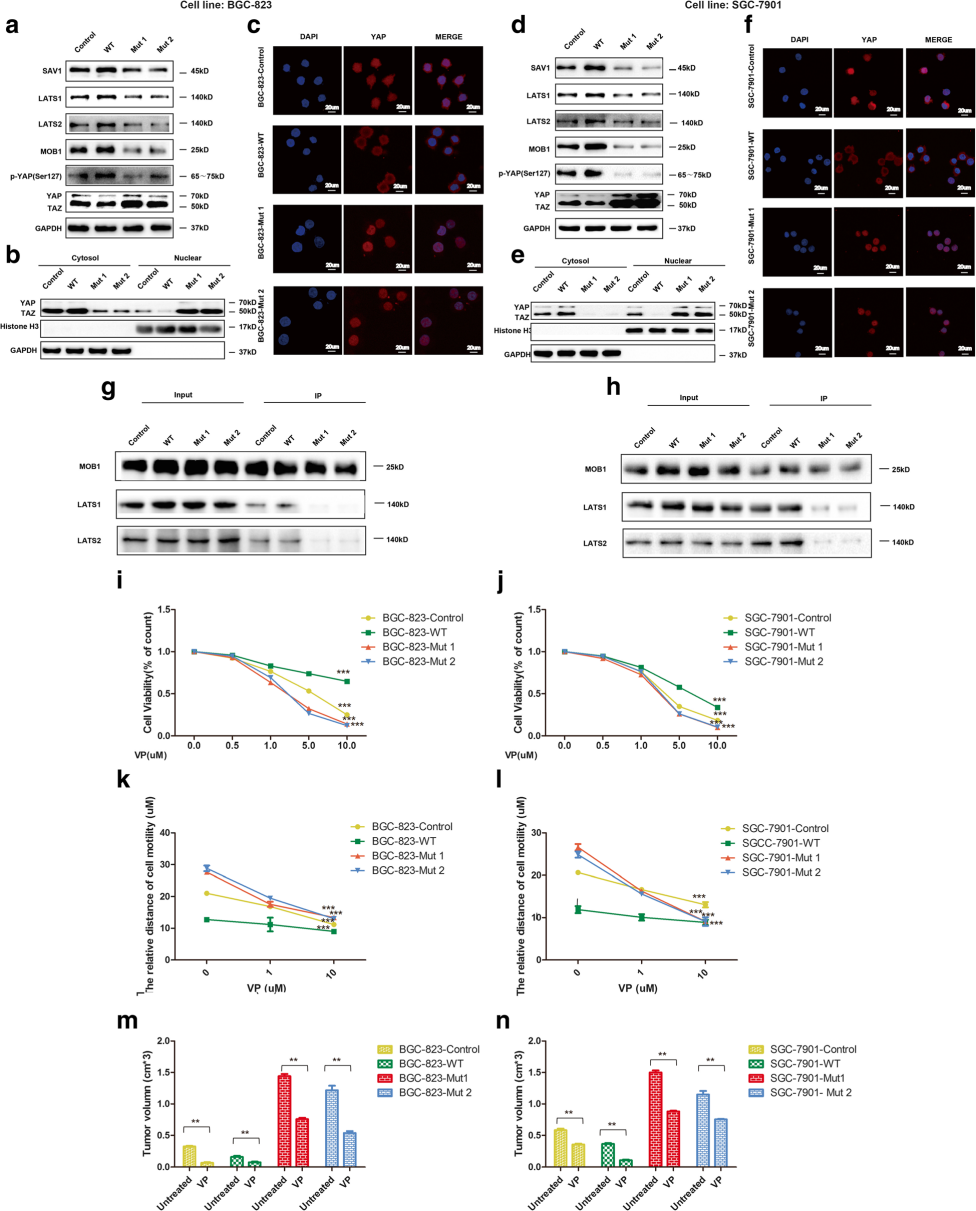
The Hippo pathway is involved in the malignant biological behaviors of GC that are induced by PTEN lipid phosphatase inactivation. **a**, **d** Immunoblot of upstream molecules and (p)-YAP/TAZ in the Hippo pathway in stable cells expressing wild-type PTEN, dominant-negative mutant type PTEN C124S (Mut 1) or G129E (Mut 2) or empty vector. **b**, **e** Immunoblot of YAP/TAZ expression levels in nuclear and cytoplasmic proteins of the stable cells. **c**, **f** Immunofluorescence analysis of YAP (red) and DAPI (blue) in stable cells. **g**, **h** Coimmunoprecipitation analysis of MOB1-LATS1/2 by incubating with an anti-MOB1 antibody in stable cells. **i**, **j** Cell viability of the cells after treatment with 0, 0.5, 1, 5, and 10 μM VP for 72 h. **k**, **l** Relative distance of cell motility after treatment with 0, 1, and 10 μM VP for 72 h. **m**, **n** Quantification of tumor volumn *, *p* < 0.05; **, *p* < 0.01

To further verify whether PTEN lipid phosphatase inactivation affects the Hippo pathway, we detected the expression of key molecules in the Hippo pathway in the stable cell lines expressing wild-type PTEN, a dominant-negative mutant PTEN (C124S and G129E) or an empty vector. The results showed that compared with the control, dominant-negative mutant PTEN significantly decreased the expression of SAV1, LATS1, LATS2, MOB1 as well as p-YAP (Ser127) and increased the expression of YAP/TAZ. In contrast, wild-type PTEN upregulated the expression of SAV1, LATS1, LATS2, MOB1 and p-YAP (Ser127) and downregulated the expression of YAP/TAZ (Fig. 4a, d).

### PTEN lipid phosphatase inactivation promotes YAP translocation

Interestingly, PTEN lipid phosphatase inactivation redistributed YAP to the nucleus, which could be observed by examining the expression levels of YAP/TAZ in the nuclear and cytoplasmic protein fractions (Fig. 4b, e). Further detection of the YAP location in stable GC cell lines through an immunofluorescence assay showed that YAP was mainly localized in the nucleus in Mut 1 and Mut 2 cells and mainly in the cytoplasm in the WT group (Fig. 4c, f).

### PTEN lipid phosphatase inactivation inhibits the binding of MOB1 to LATS1/2

We subsequently tested for a physical interaction between the upstream regulator MOB1 with LATS1 or LATS2 by coimmunoprecipitation with anti-MOB1 antibody in the stable cells. Surprisingly, we found that compared with the control, dominant-negative mutant PTEN (C124S and G129E) decreased the MOB1–LATS1/2 interaction, whereas the stable cell lines expressing wild-type PTEN showed increased MOB1–LATS1/2 binding (Fig. 4g, h).

### VP inhibits the proliferation and migration of GC cells induced by PTEN lipid phosphatase inactivation

To further identify how Hippo/YAP contributes to the gastric tumorigenesis mediated by PTEN lipid phosphatase inactivation, we used VP, which effectively disrupts YAP-TEAD-driven transcription, at different concentrations and detected GC cell proliferation and migration. Interestingly, after treatment with VP for 72 h, cell proliferation and motility were significantly inhibited in a dose-dependent manner in the stable cell lines, and this trend was particularly obvious in the stable cell lines expressing dominant-negative mutant PTEN (Mut 1 and Mut 2), which rapidly exhibited decreased cell numbers and motility distances (Fig. 4i–l). To further confirm effect of VP on GC proliferation in vivo, the xenograft model was adopted using nude mice as previously indicated. Then VP (100 mg/kg) was injected into the mouse intraperitoneally every 3 day after tumor constructed, while mouse in untreated group using PBS. Mouse were sacrificed after five doses of VP and tumor volumes were measured. It showed that the tumors were dramatically smaller in VP-treated group than the untreated. (Fig. 4m, n; see also Additional file 4 for more detail). Overall, these data suggest that the Hippo pathway is involved in the malignant biological behaviors of GC that are induced by PTEN lipid phosphatase inactivation.

### PTEN lipid phosphatase inactivation activates the PI3K/Akt pathway in a hippo pathway-dependent manner

**Fig. 5:**
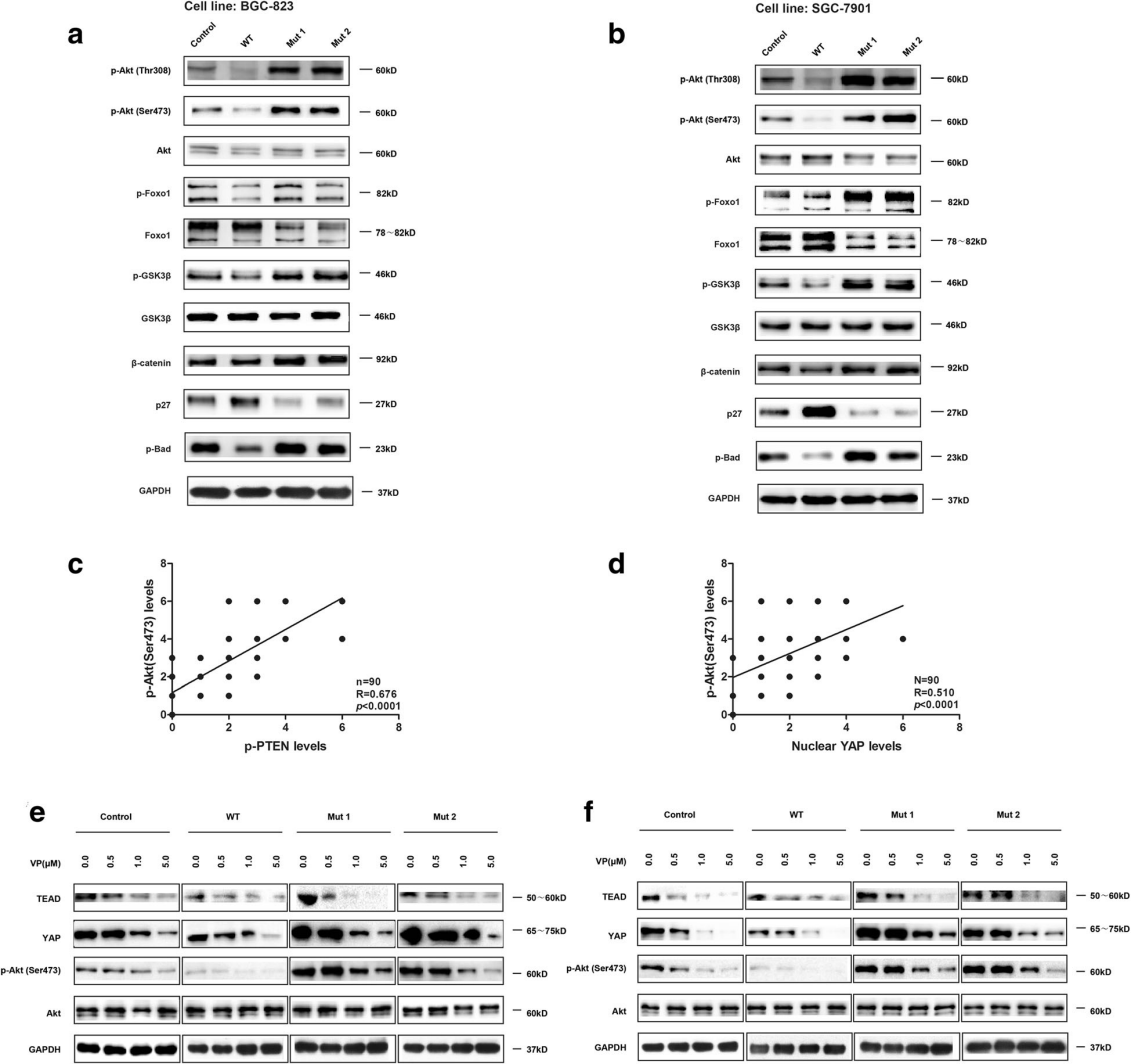
PTEN lipid phosphatase inactivation activates the PI3K/Akt pathway through a mechanism dependent on the Hippo pathway. **a**, **b** The expression of key proteins in the PI3K/Akt pathway was detected in stable cell lines. **c**, **d** Association between p-Akt (Ser473) and p-PTEN or nuclear YAP in GC specimens. **e**, **f** The expression of Akt and p-Akt was detected after treatment with 0, 0.5, 1, and 5 μM VP

To verify the effect of PTEN lipid phosphatase inactivation on the PI3K/Akt pathway, we detected the expression of key proteins in the PI3K/Akt pathway in the stable cells. The results showed that PTEN lipid phosphatase inactivation activated the PI3K/Akt pathway with increased expression of p-Akt (Ser308, Ser473), p-FoxO1/FoxO1, p-GSK-3β, β-catenin, and p-Bad and decreased p27 expression. In contrast, the robust phosphatase activity in the WT group had the opposite effect on the expression of these molecules (Fig. 5a, b). We also detected the p-Akt (Ser473) level in the cohort of 90 GC samples indicated previously by IHC and analysed correlation between p-Akt (Ser473) and p-PTEN or nuclear YAP. It showed that p-Akt (Ser473) expression was positively correlated with expression of p-PTEN and nuclear YAP (Fig. 5c, d). Moreover, after treatment with VP at different doses, a concentration-dependent reduction of p-Akt in the stable GC cell lines was evident (Fig. 5e, f).

## Discussion

PTEN deletion has been recognized as a driver of many sporadic cancers in the clinic [26–31]. PTEN has dual activity of a protein phosphatase and a lipid phosphatase and can thus regulate a complex set of signaling pathways. However, the substitution effect of protein phosphatase activity remains unclear. Therefore, we investigated the role of the loss of PTEN phosphatase and lipid phosphatase functions in GC development. We established stable cell lines that expressed wild-type PTEN (with robust phosphatase activity), dominant-negative mutant PTEN C124S (loss of complete phosphatase activity), or G129E (lacks only lipid phosphatase activity) to conduct functional and mechanistic studies in vitro and in vivo. Our results showed that PTEN lipid phosphatase inactivation increased the malignant biological behavior of GC cells, especially cell proliferation and migration. The analysis of in vivo tumor growth in nude mice also confirmed that PTEN lipid phosphatase inactivation was positively related to GC progression. Another study on transgenic mice also showed that compared with heterozygous mice carrying one wild-type allele and one null allele, mice carrying both a wild-type PTEN allele and a mutant PTEN (C124S/G129E) allele had a higher risk of breast cancer [22, 32]. These findings suggest the significance of PTEN lipid phosphatase inactivation on tumorigenesis.

PTEN phosphorylation at Ser380/Thr382/383 can deprive PTEN of phosphatase activity and maintain PTEN stability, similarly to dominant-negative mutant PTEN (C124S and G129E). Therefore, in our study, we detected the expression of p-PTEN (Ser380/Thr382/383) in human tissues to reflect the role of PTEN phosphatase inactivation in GC and found that the expression of p-PTEN in GC tissues was significantly higher than that in ANTT. One study showed that PTEN inactivation may occur as a relatively late event during cancer development [33]. In this study, we found that p-PTEN expression was positively correlated with the depth of tumor invasion, lymph node metastasis, clinical stage and a poor prognosis in GC patients and is an independent prognostic factor for GC. Based on these findings, p-PTEN (PTEN phosphatase inactivation) is expected to become an effective biomarker for predicting the risk of GC occurrence and prognosis.

To date, the known functions of the Hippo signaling pathway are mainly the following: (1) to regulate the size and growth of tissues and organs, (2) to regulate cell proliferation and apoptosis, (3) to regulate the epithelial-mesenchymal transition and intercellular contact, and (4) to maintain stem cell self-renewal and versatility [34]. Dysregulation of the Hippo pathway may cause uncontrolled phenotypic changes, which result in disease [35]. YAP is the key effector downstream of the Hippo pathway. However, there are some controversies regarding the suppression or promoting role of YAP in cancer. Barry et al. [36] found that YAP expression was decreased in colon cancer patients, thereby inhibiting the Wnt signaling pathway in the colon. However, Wang et al. [37] reported that 53.5% of colon cancer patients show YAP overexpression, and this overexpression was found to be associated with lymph node metastasis, TNM staging, and a short overall survival rate. In addition, another study on breast cancer found that YAP showed higher nuclear expression in normal breast tissues and was absent in breast cancer tissues [38]. Furthermore, although the role of YAP in human glioma cells is difficult to judge, there is an indication for YAP overexpression [39]. These reports suggest that YAP may play a dual role in suppressing and promoting cancer in these types of tumor.

What is the exact role of YAP in gastric cancer? To address this question, we detected YAP expression in 90 cases of GC and their paired ANTTs and found that YAP expression was significantly higher in GC tissues than in ANTTs. The total and nuclear YAP levels were positively correlated with the tumor size, depth of tumor invasion, lymph node metastasis, clinical stage and poor prognosis in GC patients, whereas the cytoplasmic YAP levels did not correlate with these clinicopathologic features. In addition, both the total and nuclear YAP levels are independent prognostic factors for GC. Based on the above-described results, it can be noted that YAP may play an important role in promoting GC development, and the localization of YAP in GC cells may lead to different risks of disease. However, YAP can exert different functions in the cytoplasm and nucleus, which depend on the tissue type. For example, a previous study found that YAP expression was elevated in cervical squamous cell carcinomas and that the cytoplasmic YAP level was positively correlated with lymph node metastasis, high-grade tissue type, and early recurrence. In contrast, nuclear YAP was overexpressed in cervical adenocarcinoma, and increased nuclear YAP led to a reduction in the overall survival rate [40]. These results suggest that YAP may serve as a potential anti-cancer target, which has important clinical significance.

However, the regulatory mechanism of Hippo/YAP remains unclear. In our study, we found that the expression levels of nuclear YAP were positively related to p-PTEN in GC tissues. Interestingly, our study also demonstrated that PTEN lipid phosphatase inactivation decreased the expression of upstream kinases in the Hippo pathway, increased YAP phosphorylation and promoted YAP translocation into the nucleus in stable GC cell lines. Furthermore, previous studies emphasized that increased binding of MOB1 with LATS1/2 promotes further activation of the upstream-kinase-activation-loop, thus increasing YAP phosphorylation and inhibiting the nuclear translocation of YAP [41, 42], also in our study, we found that PTEN lipid phosphatase inactivation abolished the MOB1-LATS1/2 complexes, which may contribute to the observed decrease in YAP phosphorylation and the enhancement of nuclear localization.

We then added VP, which can effectively decrease YAP/TEAD expression and disrupt YAP-TEAD-driven transcription, to the stable cell lines to determine how Hippo/YAP contributes to the gastric tumorigenesis mediated by PTEN lipid phosphatase inactivation. We found that cell proliferation and motility were significantly inhibited in a dose-dependent manner in the stable cell lines, especially in the cells expressing dominant-negative mutant PTEN. The analysis of in vivo tumor growth in nude mice also confirmed the important role of Hippo/YAP in PTEN lipid phosphatase inactivation induced GC progression. In summary, PTEN lipid phosphatase inactivation regulates the Hippo signaling pathway, reduces YAP phosphorylation, promotes YAP nuclear translocation, and leads to an enhanced synergistic effect of YAP-TEAD, thus inducing GC proliferation and migration.

The PI3K/Akt signaling pathway is important in controlling cell survival, promoting proliferation and inhibiting apoptosis [20]. Our studies demonstrated that PTEN lipid phosphatase inactivation activated PI3K/Akt and regulated downstream molecules, such as FoxO, GSK-3β, β-catenin, p27 and Bad. And also p-Akt (Ser473) expression was positively correlated with expression of p-PTEN in human GC tissues. Because Hippo/YAP and PI3K/Akt are both involved in the regulation of PTEN lipid phosphatase activity, we further explored their link. Interestingly, we found expression of p-Akt (Ser473) was positively correlated with that of nuclear YAP in human GC tissues. Strikingly, we further found that VP could decrease Akt activation in a dose-dependent manner. This finding was consistent with previous studies, as some reports have confirmed the synergistic effects of Hippo/YAP and P13K/Akt on cell proliferation and apoptosis. Some scholars found that Yki, a homolog of YAP in Drosophila, positively regulates the expression and activity of Akt, and activation of the Hippo signaling pathway can reduce Akt expression [43]. Tumaneng et al. [23] found that overexpression of YAP increased phosphorylation of Akt and conversely, p-Akt was reduced in YAP knockdown cells. In addition, a study on medulloblastoma found that YAP could increase the radioresistance of tumor cells by inducing the expression of insulin-like growth factor and Akt activation, thereby promoting the growth of tumor cells after radiotherapy [44]. Another in vivo study has shown that the binding of YAP-TEAD directly promotes the transcription of P13K, thereby activating the PI3K/Akt signaling pathway and promoting myocardial cell proliferation in rats [45, 46].

## Conclusions

**Fig. 6:**
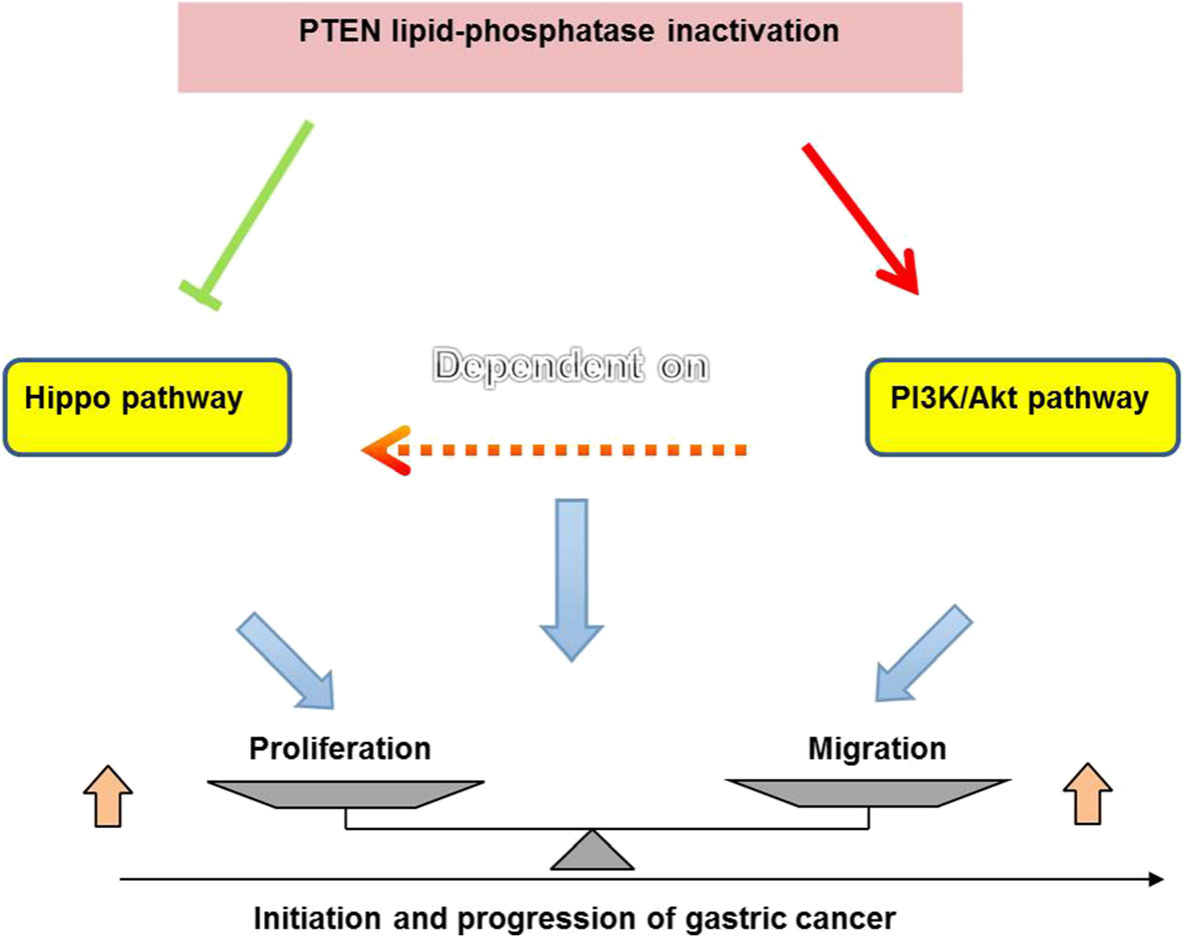
A schematic model of the role and underlying mechanism of PTEN lipid phosphatase inactivation in GC development. PTEN lipid phosphatase inactivation negatively regulates the Hippo signaling pathway and activates the PI3K/Akt signaling pathway, leading to enhanced GC proliferation and migration and resulting in gastric tumorigenesis and development

In summary, we elucidated a schematic model for the role of PTEN lipid phosphatase inactivation in GC development (Fig. 6). Taken together, the results of this study indicate that PTEN lipid phosphatase inactivation negatively regulates the Hippo signaling pathway and activates the PI3K/Akt signaling pathway, leading to enhanced GC proliferation and migration, which contribute to gastric tumorigenesis and development. This new molecular mechanism may contribute to potential benefits associated with early diagnosis and gene therapy-based treatment strategies.

## Additional files

Additional file 1: **Table S1.** p-PTEN expression in GC tissues and ANTTs. (DOCX 18 kb)

Additional file 2: **Figure S1.** PTEN and p-PTEN are overexpressed in BGC-823 and SGC-7901 cells expressing wild-type (WT) or dominant-negative mutant PTEN C124S (Mut 1) or G129E (Mut 2). (DOCX 152 kb)

Additional file 3: **Table S2.** YAP expression in GC tissues and ANTTs. (DOCX 13 kb)

Additional file 4: **Figure S2.** VP (100 mg/kg) was injected into the xenograft mouse intraperitoneally every 3 day after tumor constructed. After five doses of VP, tumor volumes were measured. Tumors from each group (*n* = 3) are shown. (DOCX 7919 kb)

## Abbreviations

FBS: Fetal bovine serum
GC: Gastric cancer
IHC: Immunohistochemistry
LATS1/2: Large tumor suppressor 1/2
MOB1: MOB kinase activator 1
MST1/2: STE20-like protein kinase 1/2
PTEN: Phosphatase and tensin homolog
SAV1: Human salvador Homozygous 1
TAZ: Transcriptional co-activator with PDZ binding motif
VP: Verteporfin
YAP: Downstream transcription coactivators, including Yes-associated protein

## References

[1] Torre LA, Bray F, Siegel RL, Ferlay J, Lortet-Tieulent J, Jemal A (2015). Global cancer statistics, 2012. CA Cancer J Clin.

[2] Chen W, Zheng R, Baade PD, Zhang S, Zeng H, Bray F (2016). Cancer statistics in China, 2015. CA Cancer J Clin.

[3] Deng N, Goh LK, Wang H, Das K, Tao J, Tan IB (2012). A comprehensive survey of genomic alterations in gastric cancer reveals systematic patterns of molecular exclusivity and co-occurrence among distinct therapeutic targets. Gut.

[4] Li J, Yen C, Liaw D, Podsypanina K, Bose S, Wang SI (1997). PTEN, a putative protein tyrosine phosphatase gene mutated in human brain, breast, and prostate cancer. Science.

[5] Zhang LL, Liu J, Lei S, Zhang J, Zhou W, Yu HG (2014). PTEN inhibits the invasion and metastasis of gastric cancer via downregulation of FAK expression. Cell Signal.

[6] Hopkins BD, Hodakoski C, Barrows D, Mense SM, Parsons RE (2014). PTEN function: the long and the short of it. Trends Biochem Sci.

[7] Filbin MG, Dabral SK, Pazyra-Murphy MF, Ramkissoon S, Kung AL, Pak E (2013). Coordinate activation of Shh and PI3K signaling in PTEN-deficient glioblastoma: new therapeutic opportunities. Nat Med.

[8] Papa A, Wan L, Bonora M, Salmena L, Song MS, Hobbs RM (2014). Cancer-associated PTEN mutants act in a dominant-negative manner to suppress PTEN protein function. Cell.

[9] Furnari FB, Huang HJ, Cavenee WK (1998). The phosphoinositol phosphatase activity of PTEN mediates a serum-sensitive G1 growth arrest in glioma cells. Cancer Res.

[10] Justice RW, Zilian O, Woods DF, Noll M, Bryant PJ (1995). The Drosophila tumor suppressor gene warts encodes a homolog of human myotonic dystrophy kinase and is required for the control of cell shape and proliferation. Genes Dev.

[11] Kim MH, Kim YK, Shin DH, Lee HJ, Shin N, Kim A (2015). Yes associated protein is a poor prognostic factor in well-differentiated lung adenocarcinoma. Int J Clin Exp Pathol.

[12] Sun L, Chen F, Shi W, Qi L, Zhao Z, Zhang J (2014). Prognostic impact of TAZ and beta-catenin expression in adenocarcinoma of the esophagogastric junction. Diagn Pathol.

[13] Yagi H, Asanoma K, Ohgami T, Ichinoe A, Sonoda K, Kato K (2016). GEP oncogene promotes cell proliferation through YAP activation in ovarian cancer. Oncogene.

[14] Liu JY, Li YH, Lin HX, Liao YJ, Mai SJ, Liu ZW (2013). Overexpression of YAP 1 contributes to progressive features and poor prognosis of human urothelial carcinoma of the bladder. BMC Cancer.

[15] Li H, Wang S, Wang G, Zhang Z, Wu X, Zhang T (2014). Yes-associated protein expression is a predictive marker for recurrence of hepatocellular carcinoma after liver transplantation. Dig Surg.

[16] Yu FX, Zhao B, Guan KL (2015). Hippo pathway in organ size control, tissue homeostasis, and Cancer. Cell.

[17] Lamar JM, Stern P, Liu H, Schindler JW, Jiang ZG, Hynes RO (2012). The hippo pathway target, YAP, promotes metastasis through its TEAD-interaction domain. Proc Natl Acad Sci U S A.

[18] Kanai F, Marignani PA, Sarbassova D, Yagi R, Hall RA, Donowitz M (2000). TAZ: a novel transcriptional co-activator regulated by interactions with 14-3-3 and PDZ domain proteins. EMBO J.

[19] Cherrett C, Furutani-Seiki M, Bagby S (2012). The hippo pathway: key interaction and catalytic domains in organ growth control, stem cell self-renewal and tissue regeneration. Essays Biochem.

[20] Yang Z, Xie C, Xu W, Liu G, Cao X, Li W (2015). Phosphorylation and inactivation of PTEN at residues Ser380/Thr382/383 induced by helicobacter pylori promotes gastric epithelial cell survival through PI3K/Akt pathway. Oncotarget.

[21] Myers MP, Pass I, Batty IH, Van der Kaay J, Stolarov JP, Hemmings BA (1998). The lipid phosphatase activity of PTEN is critical for its tumor supressor function. Proc Natl Acad Sci U S A.

[22] Wang H, Karikomi M, Naidu S, Rajmohan R, Caserta E, Chen HZ (2010). Allele-specific tumor spectrum in pten knockin mice. Proc Natl Acad Sci U S A.

[23] Tumaneng K, Schlegelmilch K, Russell RC, Yimlamai D, Basnet H, Mahadevan N (2012). YAP mediates crosstalk between the hippo and PI(3)K-TOR pathways by suppressing PTEN via miR-29. Nat Cell Biol.

[24] Garcia-Rendueles ME, Ricarte-Filho JC, Untch BR, Landa I, Knauf JA, Voza F (2015). NF2 loss promotes oncogenic RAS-induced thyroid cancers via YAP-dependent transactivation of RAS proteins and sensitizes them to MEK inhibition. Cancer Discov.

[25] Yang Z, Yuan XG, Chen J, Luo SW, Luo ZJ, Lu NH (2013). Reduced expression of PTEN and increased PTEN phosphorylation at residue Ser380 in gastric cancer tissues: a novel mechanism of PTEN inactivation. Clin Res Hepatol Gastroenterol.

[26] Song MS, Salmena L, Pandolfi PP (2012). The functions and regulation of the PTEN tumour suppressor. Nat Rev Mol Cell Biol.

[27] Yuan TL, Cantley LC (2008). PI3K pathway alterations in cancer: variations on a theme. Oncogene.

[28] Li DM, Sun H (1997). TEP1, encoded by a candidate tumor suppressor locus, is a novel protein tyrosine phosphatase regulated by transforming growth factor beta. Cancer Res.

[29] Steck PA, Pershouse MA, Jasser SA, Yung WK, Lin H, Ligon AH (1997). Identification of a candidate tumour suppressor gene, MMAC1, at chromosome 10q23.3 that is mutated in multiple advanced cancers. Nat Genet.

[30] Hollander MC, Blumenthal GM, Dennis PA (2011). PTEN loss in the continuum of common cancers, rare syndromes and mouse models. Nat Rev Cancer.

[31] Nassif NT, Lobo GP, Wu X, Henderson CJ, Morrison CD, Eng C (2004). PTEN mutations are common in sporadic microsatellite stable colorectal cancer. Oncogene.

[32] Rodriguez-Escudero I, Oliver MD, Andres-Pons A, Molina M, Cid VJ, Pulido R (2011). A comprehensive functional analysis of PTEN mutations: implications in tumor- and autism-related syndromes. Hum Mol Genet.

[33] Kang YH, Lee HS, Kim WH (2002). Promoter methylation and silencing of PTEN in gastric carcinoma. Lab Investig.

[34] Hilman D, Gat U (2011). The evolutionary history of YAP and the hippo/YAP pathway. Mol Biol Evol.

[35] Yu FX, Zhao B, Panupinthu N, Jewell JL, Lian I, Wang LH (2012). Regulation of the hippo-YAP pathway by G-protein-coupled receptor signaling. Cell.

[36] Barry ER, Morikawa T, Butler BL, Shrestha K, de la Rosa R, Yan KS (2013). Restriction of intestinal stem cell expansion and the regenerative response by YAP. Nature.

[37] Wang Y, Xie C, Li Q, Xu K, Wang E (2013). Clinical and prognostic significance of yes-associated protein in colorectal cancer. Tumour Biol.

[38] Tufail R, Jorda M, Zhao W, Reis I, Nawaz Z (2012). Loss of yes-associated protein (YAP) expression is associated with estrogen and progesterone receptors negativity in invasive breast carcinomas. Breast Cancer Res Treat.

[39] Liu YC, Wang YZ (2015). Role of yes-associated protein 1 in gliomas: pathologic and therapeutic aspects. Tumour Biol.

[40] Liu T, Liu Y, Gao H, Meng F, Yang S, Lou G (2013). Clinical significance of yes-associated protein overexpression in cervical carcinoma: the differential effects based on histotypes. Int J Gynecol Cancer.

[41] Praskova M, Xia F, Avruch J (2008). MOBKL1A/MOBKL1B phosphorylation by MST1 and MST2 inhibits cell proliferation. Curr Biol.

[42] Kim M, Kim M, Lee S, Kuninaka S, Saya H, Lee H (2013). cAMP/PKA signalling reinforces the LATS-YAP pathway to fully suppress YAP in response to actin cytoskeletal changes. EMBO J.

[43] Ye X, Deng Y, Lai ZC (2012). Akt is negatively regulated by hippo signaling for growth inhibition in Drosophila. Dev Biol.

[44] Fernandez LA, Squatrito M, Northcott P, Awan A, Holland EC, Taylor MD (2012). Oncogenic YAP promotes radioresistance and genomic instability in medulloblastoma through IGF2-mediated Akt activation. Oncogene.

[45] Del Re DP, Yang Y, Nakano N, Cho J, Zhai P, Yamamoto T (2013). Yes-associated protein isoform 1 (yap 1) promotes cardiomyocyte survival and growth to protect against myocardial ischemic injury. J Biol Chem.

[46] Lin Z, Zhou P, von Gise A, Gu F, Ma Q, Chen J (2015). Pi3kcb links hippo-YAP and PI3K-AKT signaling pathways to promote cardiomyocyte proliferation and survival. Circ Res.

