# Supplementary material for: PTEN lipid phosphatase inactivation links the hippo and PI3K/Akt pathways to induce gastric tumorigenesis
Source: J Exp Clin Cancer Res. 2018 Aug 22;37:198. doi: 10.1186/s13046-018-0795-2 (PMC6104022; doi:10.1186/s13046-018-0795-2)
Supplement: Supplementary file 1 — Table S1. p-PTEN expression in GC tissues and ANTTs. (DOCX 18 kb) [file 13046_2018_795_MOESM1_ESM.docx]

**Additional file 1：**Table S1**.** p-PTEN expression in GC tissues and ANTTs

| Variables | p-PTEN expression | | |  |
| --- | --- | --- | --- | --- |
|  | All cases  (n=180) | Low expression  (n=121) | High expression  (n=59) | *p* value |
| GC | 90 | 46 | 44 | 0.000 |
| ANTT | 90 | 75 | 15 |  |
