# Supplementary material for: PTEN lipid phosphatase inactivation links the hippo and PI3K/Akt pathways to induce gastric tumorigenesis
Source: J Exp Clin Cancer Res. 2018 Aug 22;37:198. doi: 10.1186/s13046-018-0795-2 (PMC6104022; doi:10.1186/s13046-018-0795-2)

**Additional file 2:** Figure S1. PTEN and p-PTEN are overexpressed in BGC-823 and SGC-7901 cells expressing wild-type (WT) or dominant-negative mutant PTEN C124S (Mut 1) or G129E (Mut 2).


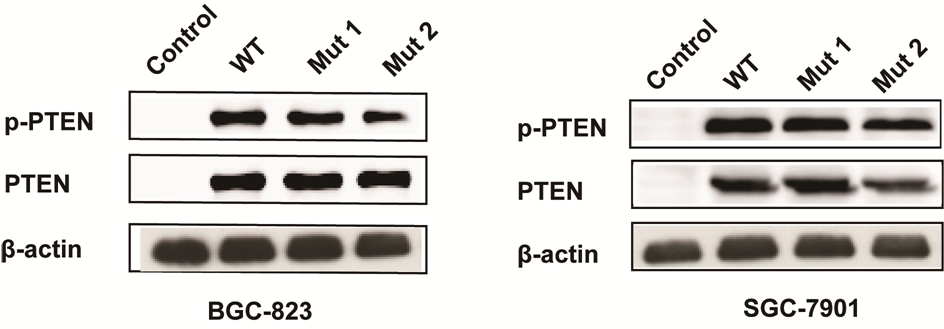

Supplement: Supplementary file 2 — Figure S1. PTEN and p-PTEN are overexpressed in BGC-823 and SGC-7901 cells expressing wild-type (WT) or dominant-negative mutant PTEN C124S (Mut 1) or G129E (Mut 2). (DOCX 152 kb) [file 13046_2018_795_MOESM2_ESM.docx]
