# Supplementary material for: PTEN lipid phosphatase inactivation links the hippo and PI3K/Akt pathways to induce gastric tumorigenesis
Source: J Exp Clin Cancer Res. 2018 Aug 22;37:198. doi: 10.1186/s13046-018-0795-2 (PMC6104022; doi:10.1186/s13046-018-0795-2)
Supplement: Supplementary file 3 — Table S2. YAP expression in GC tissues and ANTTs. (DOCX 13 kb) [file 13046_2018_795_MOESM3_ESM.docx]

**Additional file 3:** Table S2. YAP expression in GC tissues and ANTTs

| Variables | YAP expression | | |  |
| --- | --- | --- | --- | --- |
|  | All cases  (n=180) | Low expression  (n=114) | High expression  (n=66) | *p* value |
| GC | 90 | 37 | 53 | <0.001 |
| ANTT | 90 | 77 | 13 |  |
