# Supplementary material for: PTEN lipid phosphatase inactivation links the hippo and PI3K/Akt pathways to induce gastric tumorigenesis
Source: J Exp Clin Cancer Res. 2018 Aug 22;37:198. doi: 10.1186/s13046-018-0795-2 (PMC6104022; doi:10.1186/s13046-018-0795-2)
Supplement: Supplementary file 4 — Figure S2. VP (100 mg/kg) was injected into the xenograft mouse intraperitoneally every 3 day after tumor constructed. After five doses of VP, tumor volumes were measured. Tumors from each group (n = 3) are shown. (DOCX 7919 kb) [file 13046_2018_795_MOESM4_ESM.docx]

**Additional file 4:** Figure S2. VP (100mg/kg) was injected into the xenograft mouse intraperitoneally every 3 day after tumor constructed. After five doses of VP, tumor volumes were measured. Tumors from each group (n=3) are shown.

**
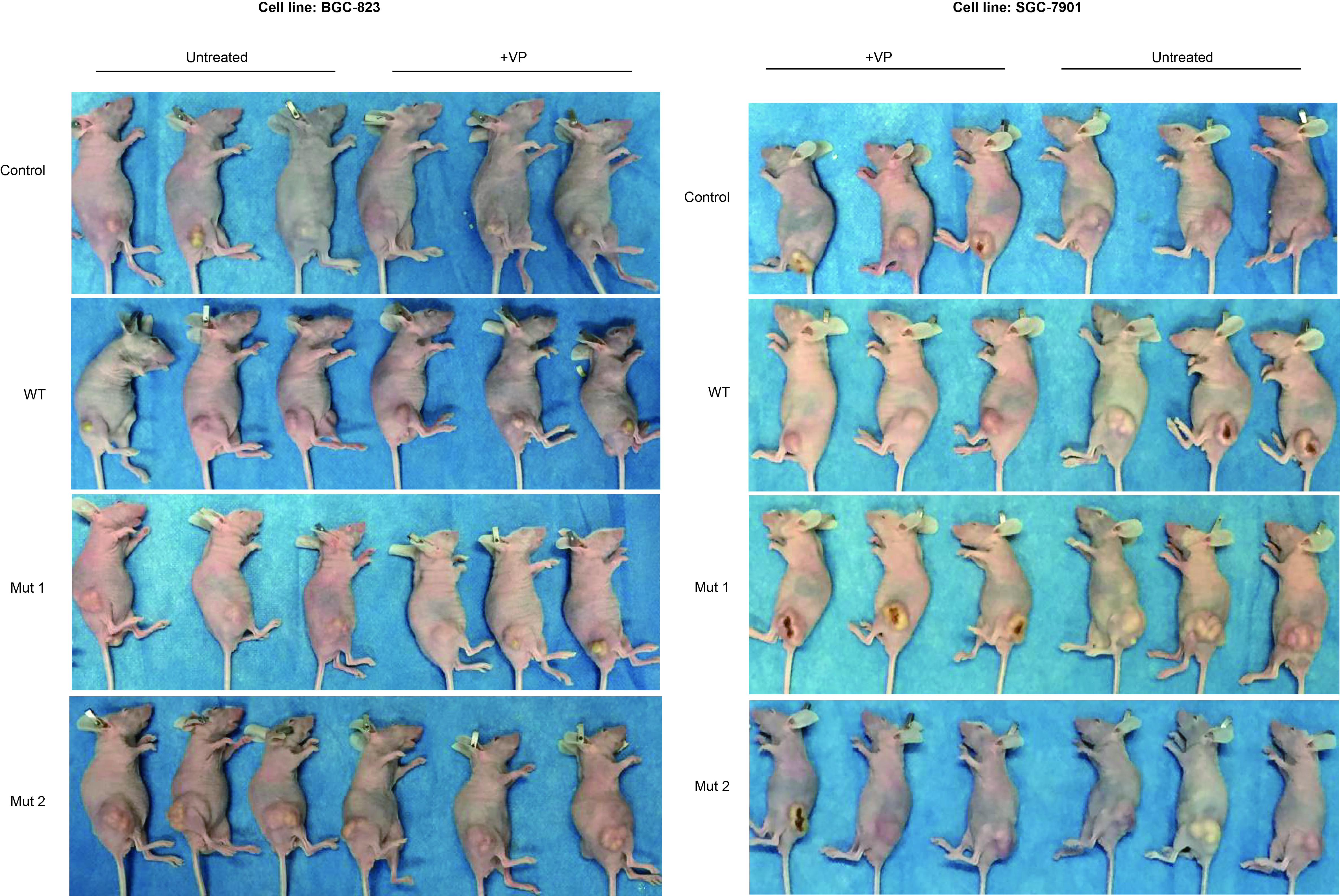
**
